# Serological and molecular identification of Reticuloendotheliosis virus (REV) in chickens in Sudan

**DOI:** 10.1002/vms3.188

**Published:** 2019-07-26

**Authors:** Shima H. Alfaki, Mohammed O. Hussien, Fadwa M. Elsheikh, Khalid M. Taha, Atif H. Elbrissi, Abdel Rahim M. El Hussein

**Affiliations:** ^1^ Central Veterinary Research Laboratory (CVRL) Animal Resources Research Corporation (ARRC) Khartoum Sudan; ^2^ Central Laboratory Ministry of Higher Education and Scientific Research Khartoum Sudan; ^3^ Atbara Veterinary Research Laboratory (AVRL) Atbara Sudan

**Keywords:** chickens, ELISA, PCR, prevalence, Reticuloendotheliosis virus (REV), Sudan

## Abstract

**Background:**

Reticuloendotheliosis virus (REV) is a gammaretrovirus that belongs to the family of *Retroviridae*. The infection can result in immunosuppression, runting syndrome, high mortality, acute reticular cell neoplasia or T‐ and/ or B‐cell lymphoma, in a variety of domestic and wild birds. The disease is widespread around the world. No related data have been reported in Sudan about the disease. The present study was conducted to determine the prevalence of REV antibodies and DNA in local and commercial breeds of chickens older than 20 weeks from June 2014 to February, 2017.

**Methods:**

A total of 460 sera samples and 150 (50 liver and 100 spleen) tissue samples were collected from local and commercial breeds of chickens older than 20 weeks and screened for anti‐REV antibodies in four states of Sudan using a commercial REV antibody ELISA test kit (IDEXX). Polymerase chain reaction (PCR) was performed to detect REV DNA in tissue samples in Khartoum State.

**Results:**

The results revealed that the overall seroprevalence of REV was 74.6% among local and commercial chicken breeds, but in commercial it was 79.5% (190/239) and 69.2% in local breeds (153/221). One hundred and fifty tissue samples of chickens (50 liver, 100 spleen) were tested using PCR for detection of REV using primer sets of the conserved region in envelope glycoprotein *(env)* gene with a band length of 850 bp. Five out of 50 (10%) liver samples were RE provirus DNA positive detected by PCR, whereas 15 out of 100 (15%) spleen samples were PCR positive. Univariate analysis revealed there was a difference (*p* ≤ 0.05) between locality and breed of chickens and seropositivity to REV.

**Conclusions:**

The prevalence of the disease was high in Sudan and more studies are needed to evaluate the epidemiology and pathogenesis of the virus.

## Abstract 2

This study aimed to investigate the prevalence of REV in chickens in Sudan using serological (ELISA) and molecular methods. The study revealed the high prevalence of the virus infection in both indigenous and exotic breeds of chicken in the country.

## Graphical abstract

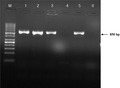

## 1 INTRODUCTION

Reticuloendotheliosis virus disease is an oncogenic, immunosuppressive, runting‐stunting syndrome of multiple avian species that is caused by Reticuloendotheliosis virus (REV), which is a gammaretrovirus with a variety of strains (Witter & Fadly, 2003). These strains include defective REV‐T, non defective REV‐A, chick syncytial virus (CSV), duck infectious anaemia virus (DIAV) and spleen necrosis virus (SNV).

REVs can cause disease in a wide range of avian hosts including chickens, turkeys, ducks, geese, pheasants, pea fowl and some other bird species (Bohls et al., 2006). The infection by REV causes immunosuppression in the infected chickens, which increases their susceptibility to concurrent or secondary bacterial or viral infection and results in poor immune responses to vaccines (Jody, Robert, Lucy, & Fadly, 2010). REV can co‐infect by their partial or complete genome with some large DNA viruses such Fowl Pox virus and Marek's disease virus (Cui, Sun, Zhang, & Meng, 2009) and also can cause contamination of a variety of poultry biologics (Garcia, Narang, Reed, & Fadly, 2003; Moore, Davis, Sato, & Yasuda, 2000).There are no obvious clinical signs seen in infected chickens. Primarily there is a runting syndrome that manifests itself as weight loss, paleness, abnormal feathering (Nakanuke disease) and immuosuppression. Tumours typically involve the liver, spleen, intestine and heart.

The virus can be transmitted horizontally by close contact or insect transmission (Ni & Cui, 2008) or vertically from infected dams to their progenies (Davidson & Braverman, 2005; Witter & Salter, 1989). The virus is also readily transmitted through contamination of vaccines and other poultry biologics (Witter & Fadly, 2003). Serological methods such as agar gel precipitin test, enzyme‐linked immunosorbent assays (ELISA) and virus neutralization are used routinely for REV diagnosis, but ELISA is more reliable and time saving (Bronzoni et al., 2005; Moshira, Abd EL‐Galiel, Asia, Hafez, & Hosny, 2016). Molecular techniques like PCR and RT‐PCR are increasingly used which are even more sensitive than ELISA (Fadly & Garcia, 2006).

There have been no previous studies that reported on REV and its associated diseases in Sudan except for one study that determined the integration of RE provirus in a fowl pox virus field isolate using PCR (Inas & Khalafalla, 2017).

The aim of this study was to detect the presence of the REV viral DNA in chickens using PCR technique and to report the seroprevalence of this disease in Sudan using ELISA technique.

## 2 MATERIALS AND METHODS

### 2.1 Sample collection

Four hundred and sixty blood samples were randomly collected from the wing vein of local and commercial breeds of apparently healthy chickens older than 20 weeks from 2–6 different flocks in four states of Sudan (Khartoum, North Kordofan, River Nile and White Nile). Sera were obtained after blood samples were centrifuged at 316 *g* for 10 min and preserved at −20°C until tested. Tissue samples (50 liver and 100 spleen) were collected from the same chicken population older than 20 weeks from chicken markets in Khartoum State. The tissue samples were collected in sterile containers and transported in ice packs and preserved at −20°C until evaluated.

### 2.2 Serological assay

The sera samples were screened for anti‐REV antibodies using the Reticuloendotheliosis virus Antibody Test Kit (IDEXX, USA) according to the manufacturer's instructions. The optical densities (OD) were measured using a microplate ELISA reader (Stat Fax 4200, USA) at 650 nm wavelength. Sera with ratios sample to positive control (S/P) of antibody titres higher than 0.5 were considered positive.

### 2.3 DNA extraction

Viral DNA was extracted from hepatic and splenic tissue samples using a commercial (innuPREP virus DNA/ RNA kit) (Analytica Jena, Germany). The extraction was done according to the protocol provided by the manufacturers and then stored at −20°C until evaluated.

### 2.4 Polymerase chain reaction (PCR)

**Figure 1:**
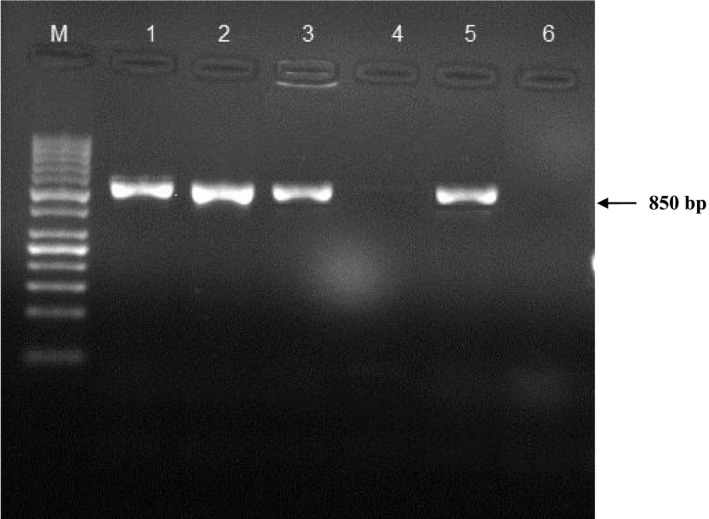
Agarose gel electrophoresis of the products amplified with PCR using the specific primers for reticuloendotheliosis virus. M; 100 bp DNA ladder, Lane 1; positive control, Lane 2, 3, 5; positive samples, Lane 4; negative sample, Lane 6; negative control

PCR amplification was carried out using primer sets for detection of REV (Forward: 5′‐GAACACAATAGGACTGG‐3′) and (Reverse: 5′‐ TTGACCTAGGGTATCCATCTC‐3′) (Hasan & Hakan, 2011). The primers amplify conserved sequences of the envelope glycoprotein (*env*) gene of REV. Amplifications were carried out at 94°C for 5 min followed by 32 cycles (94°C for 1 min, 60°C for 1 min and 72°C for 1 min) and a final extension at 72°C for 5 min. Finally, the PCR products were subjected to electrophoresis in 1% agarose gel, stained with ethidium bromide and visualized under ultraviolet (UV) light. The length of the amplicon was approximately 850 bp (Figure 1).

### 2.5 Statistical analysis

Risk factors with more than two categorical levels such as state and breed were tested individually using univariate logistic regression. Binary logistic regression was performed to test the significance of the variables in the model and to test the significance revealed by the univariate analysis. Statistical analysis was performed using SPSS version 20 (SPSS Inc., Chicago) Significance was defined as *p < *0.05.

## 3 RESULTS

The seroprevalence of REV was estimated to 69.2% (153/221) for local chicken breeds and 79.5% (190/239) for commercial breeds. The prevalence in local chickens ranged between 23.5% in White Nile State in South Sudan and 85% in River Nile State in northern Sudan (*p = *0.000) (Table 1).

**Table 1:** Univariate analysis for the association of selected risk factors on positivity of REV in chickens in Sudan during the period June 2014–February 2017

Variable	Positive/tested (prevalence% ± *SE*)		*p‐value*
	Local	Commercial	
State			
Khartoum	10/29 (34.5% ± 0.1)	190/239 (79.5% ± 0.1)	<0.001*
North Kordofan	9/22 (40.9% ± 0.1)	—	
River Nile	130/153 (85.0% ± 0.1)	—	
White Nile	4/17 (23.5% ± 0.1)	—	
Total	153/221 (69.2% ± 0.1)	190/239 (79.5% ± 0.1)	0.006*
Tissue type			
Liver	0/25 (00.0 ± 0.0)	5/25 (20.0% ± 0.1)	0.018*
Spleen	1/25 (4.0% ± 0.0)	14/75 (18.7% ± 0.1)	0.062
Total	1/50 (2.0% ± 0.0)	19/100 (19.0% ± 0.0)	

* *p‐value* ≤ 0.05 is significant.

With regard to local breed, only one splenic sample (4%) was PCR positive, while five out of 25 (20%) liver tissue samples (*p = *0.018) and 14 out of 75 (18.7%) spleen tissue samples (*p = *0.062) were positive when compared with commercial breeds. The overall prevalence of REV DNA in spleen and liver was 15% and 10%, respectively (Table 1).

There was a difference (*p ≤ *0.05) regarding locality and breed on seroprevalence. Influence of breed was detected by PCR results for REV in tissue samples collected from chicken from local markets in Khartoum State (Table 1).

## 4 DISCUSSION

Although REV is ubiquitous and the disease is very common in chickens and other birds, there are meagre studies and data about the disease in Sudan. This may be due to scant and subclinical signs or that cases are chronic and easily overlooked.

In the present study, the overall prevalence of antibodies from commercial and local breed of chickens was 74.6%, which was lower than prevalence in a study evaluating cross‐bred chickens on a farm in Delta Egypt (83.8%) (Moshira et al., 2016) and in layer chicken in Taiwan which was 85% (Wan‐Hsin, Yuan‐Pin, & Ching–Ho W., 2006). However, it was higher than that reported in China Native chicken flock (32.2%) (Peng et al., 2012). The differences in prevalence between countries may be due to the different strains, sample size and test conditions in these countries.

There was an association between breed and seropositivity of REV in chickens. The prevalence of antibodies in commercial breed was higher than in local breeds, which may be attributed to the application of contaminated vaccines such as Fowl Pox Vaccine (FPV) which were confirmed to be contaminated with REV in the current study (personal communication). These vaccines were administered to commercial chickens, but not to local chicken breeds, which are not usually vaccinated.

The origin of chicken (state) influenced seropositivity of REV in the current study. The difference in prevalence between states may be attributed to environmental differences between geographical areas and topographical reasons which affect the types of vectors present in the area and consequently efficiency of virus transmission. In addition, types and varieties of chicken with different susceptibilities or resistance may vary from one area to another.

PCR appears to be the method of choice for the diagnosis of avian oncogenic viruses as it is one of the most sensitive methods which can be applied for differential diagnosis (Davidson & Braverman, 2005). In the current study, the prevalence of positive samples by PCR for spleen and liver tissue samples were 15% and 10% respectively, which was approximately similar to prevalence of the same tissues from layer chickens in India (Sathhish, Kurunchi, & Stalin, 2015).

This study demonstrated an association between liver tissue samples from commercial breeds and positive PCR results for REV i.e. all of the liver positive samples were from commercial breeds. This may reflect a higher dose of the virus received through the contamination of fowl pox vaccines with REV.

Finally, to the best of our knowledge this is the first serological and molecular detection of REV in chickens in Sudan.

It could be concluded that the prevalence of REV (74.6%) was high in Sudan; although there were no clinical signs seen in the sampled affected chickens. However, the virus is known to suppress natural immunity and subsequent response to vaccines and increase susceptibility to concurrent diseases. Therefore, further epidemiological studies, particularly regarding transmission and control of the disease are important.

## CONFLICT OF INTEREST

The authors declare that they have no conflict of interest.

## ETHICAL STATEMENT

This study was approved by the ethics committee of the Central Veterinary Research Laboratory (CVRL), Animal Resources Research Corporation (ARRC). Sample collection was carried out according to the animal welfare code of Sudan.
